# Nursing in the Bethlem Royal Hospital

**Published:** 1921-01-08

**Authors:** 


### Nursing in the Bethlem Royal Hospital.

One of the few institutions in which no difficulty is experienced in getting probationers is the Bethlem Royal Hospital. The scale of pay is far in excess of that usually accorded to learners, and it would seem that this outweighs with the average woman seeking employment the conditions of service as regards hours on duty, which are a good deal heavier than those required from probationers in general hospitals. Candidates are received between the ages of nineteen and thirty, and serve a six months' probation before appointment. The commencing salary is £1 12s. per week, rising by increment of 1s. 7d. per week per annum to £1 19s. 11d. per week. A war bonus amounting to 15s. a week is also given. No allowance in kind, with the sole exception of uniform, is made to any member of the nursing staff, the remuneration being calculated entirely on a cash basis. Accordingly from the above totals it is necessary to deduct a charge of 7s. 9d. per week for lodging and washing and 14s. for board. The result works out at a salary, including the usual emoluments, of £65 a year. This is very handsome pay for young women, totally untrained, at the outset of their career. An increase of 2s. a week is given on obtaining the Nursing Certificate of the Medico-Psychological Association, a further increase of 2s. a week on appointment as staff nurse, and again of 3s. 3d. per week on appointment as sister. The night sister is paid an additional 2s. a week, while so employed, and there are other grants for special length of service, etc.

The hours on duty are admittedly far too long. We understand that they are considerably over sixty at present, and in some instances amount to seventy. Even under a new regulation, which will become operative as soon as the necessary additional appointments to staff are made, they are to stand at sixty per week, inclusive of six and a-quarter hours for meals. A fifty-six-hour week, not to mention forty-eight hours even in a whisper, would entail drastic changes. Whether it would not be for the advantage of the junior staff to receive less pay and more leisure is a matter which the governing body will have to consider very carefully in the near future.

An excellent operating-theatre has been added to the hospital and a full *x*-ray apparatus was installed recently. We cannot but think it very regrettable that there is no fully certificated general nurse or sister on the staff. The trend in modern mental hospitals is more and more towards assimilation with general hospital conditions, particularly in the cases requiring surgical treatment. Fully-trained nurses are received in the hospital for a small charge for special courses of mental training, but are not admitted to the staff.

The conditions under which the nurses live are of a nature to alleviate their long hours on duty. They have comfortable little separate bedrooms, and the wide and pleasant corridors in which the greater part of their duties is performed, the fine refectory, and the central hall, where music and dancing take place, all react for good. The tone of humanity and kindly feeling which characterises the institution and produces on the most perturbed brains a calming and restorative influence are the outcome of years of successful administration under medical and nursing officers deeply imbued with a high sense of their profession.

